# Supplementary material for: Biochar’s role in improving pakchoi quality and microbial community structure in rhizosphere soil
Source: PeerJ. 2024 Mar 18;12:e16733. doi: 10.7717/peerj.16733 (PMC10956520; doi:10.7717/peerj.16733)
Supplement: Supplemental Information 1 [file peerj-12-16733-s001.pdf]

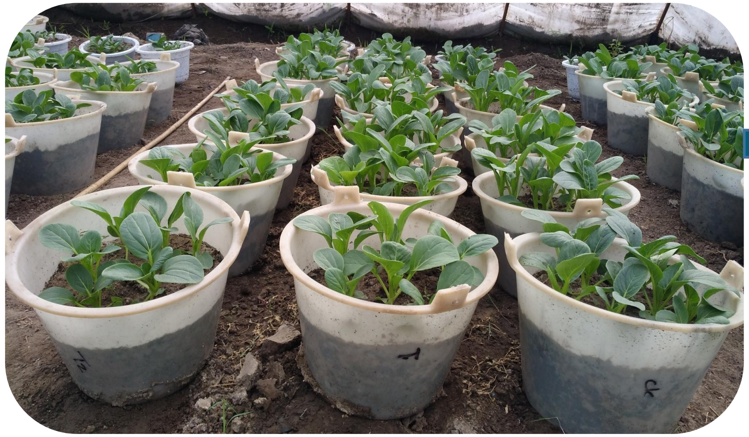

Cultivation of plant materials

Grow for 30 days

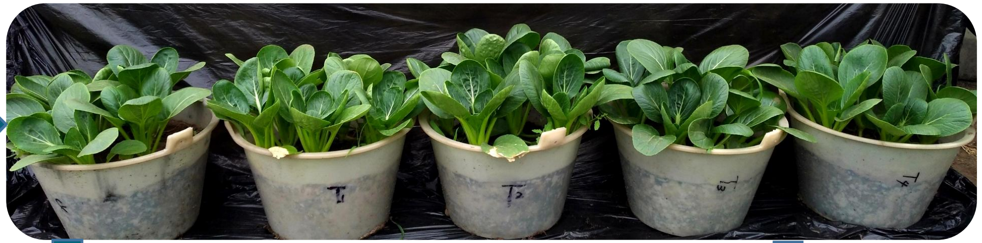

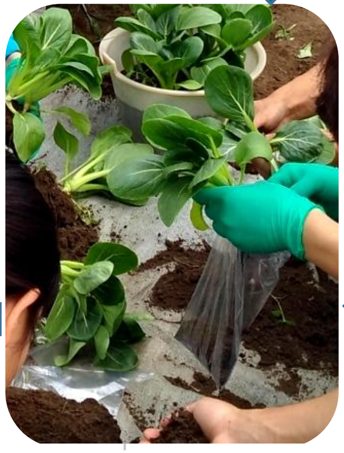

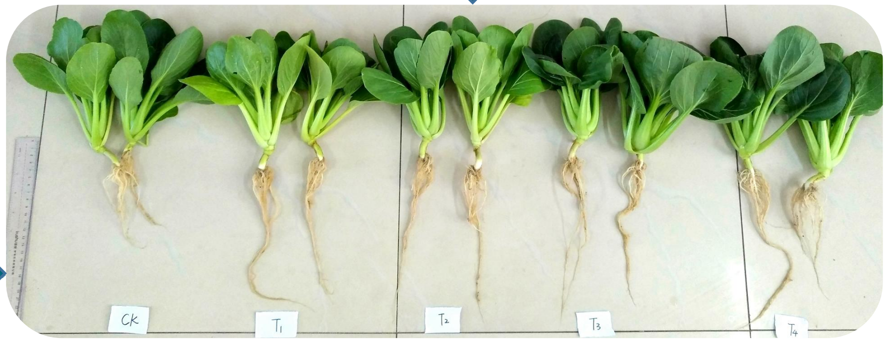

Investigation of biomass

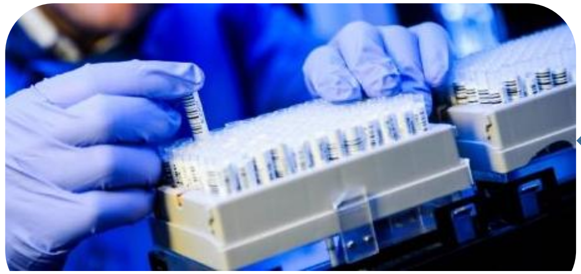

soil DNA extraction

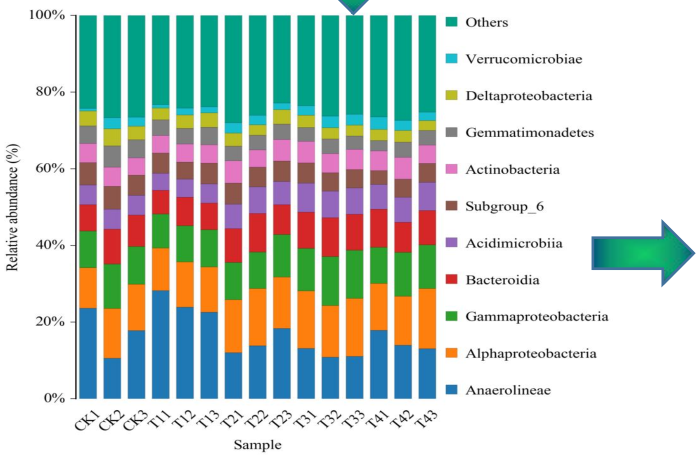

Analysis of soil microbial diversity

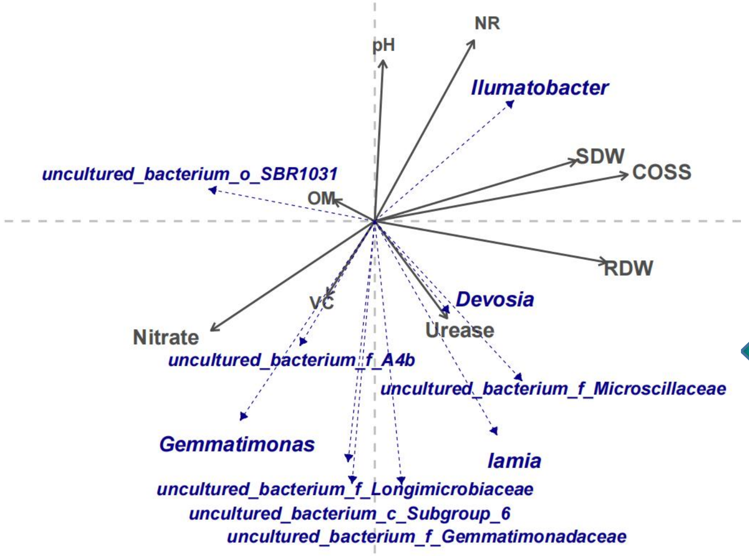

Redundancy analysis

Table 3. Effects of different cultivation modes on the quality of Chinese cabbage.

Treatments	Soluble sugar (mg·100g^{-1})	Soluble protein (mg·g^{-1})	VC (mg·100g^{-1})	Cellulose (%)	Nitrate (mg·kg^{-1})	Nitrite reductase [μg·g^{-1}·h^{-1})]
CK	25.76±1.90b	1.44±0.02b	4.32±0.54b	1.70±0.07d	286.93±5.86b	50.79±1.62b
T1	41.01±1.15a	1.55±0.05b	3.06±0.31b	2.58±0.10c	257.71±0.59c	57.54±5.03b
T2	39.18±2.33a	1.65±0.08a	5.50±0.41a	3.96±0.25b	225.67±0.44d	75.89±3.01a
T3	14.46±0.48d	1.56±0.12b	6.30±1.38a	4.56±0.18a	301.48±0.69a	88.86±4.01a
T4	20.01±0.69c	1.54±0.06b	5.40±1.08a	2.89±0.16c	288.08±9.01b	86.84±1.05a

Quality of measurement
